# The Progression of Liver Fibrosis Is Related with Overexpression of the miR-199 and 200 Families

**DOI:** 10.1371/journal.pone.0016081

**Published:** 2011-01-24

**Authors:** Yoshiki Murakami, Hidenori Toyoda, Masami Tanaka, Masahiko Kuroda, Yoshinori Harada, Fumihiko Matsuda, Atsushi Tajima, Nobuyoshi Kosaka, Takahiro Ochiya, Kunitada Shimotohno

**Affiliations:** 1 Center for Genomic Medicine, Kyoto University Graduate School of Medicine, Kyoto, Japan; 2 Department of Gastroenterology, Ogaki Municipal Hospital, Ogaki, Japan; 3 Department of Molecular Pathology, Tokyo Medical University, Tokyo, Japan; 4 Department of Pathology and Cell Regulation, Kyoto Prefectural University of Medicine, Kyoto, Japan; 5 Department of Molecular Life Science, Tokai University School of Medicine, Isehara, Japan; 6 Division of Molecular and Cellular Medicine, National Cancer Center Research Institute, Tokyo, Japan; 7 Research Institute, Chiba Institute of Technology, Narashino, Japan; Baylor College of Medicine, United States of America

## Abstract

**Background:**

Chronic hepatitis C (CH) can develop into liver cirrhosis (LC) and hepatocellular carcinoma (HCC). Liver fibrosis and HCC development are strongly correlated, but there is no effective treatment against fibrosis because the critical mechanism of progression of liver fibrosis is not fully understood. microRNAs (miRNAs) are now essential to the molecular mechanisms of several biological processes. In order to clarify how the aberrant expression of miRNAs participates in development of the liver fibrosis, we analyzed the liver fibrosis in mouse liver fibrosis model and human clinical samples.

**Methodology:**

In a CCL_4_-induced mouse liver fibrosis model, we compared the miRNA expression profile from CCL_4_ and olive oil administrated liver specimens on 4, 6, and 8 weeks. We also measured expression profiles of human miRNAs in the liver biopsy specimens from 105 CH type C patients without a history of anti-viral therapy.

**Principle Findings:**

Eleven mouse miRNAs were significantly elevated in progressed liver fibrosis relative to control. By using a large amount of human material in CH analysis, we determined the miRNA expression pattern according to the grade of liver fibrosis. We detected several human miRNAs whose expression levels were correlated with the degree of progression of liver fibrosis. In both the mouse and human studies, the expression levels of miR-199a, 199a*, 200a, and 200b were positively and significantly correlated to the progressed liver fibrosis. The expression level of fibrosis related genes in hepatic stellate cells (HSC), were significantly increased by overexpression of these miRNAs.

**Conclusion:**

Four miRNAs are tightly related to the grade of liver fibrosis in both human and mouse was shown. This information may uncover the critical mechanism of progression of liver fibrosis. miRNA expression profiling has potential for diagnostic and therapeutic applications.

## Introduction

Chronic viral hepatitis is a major risk factor for hepatocellular carcinoma (HCC) [1]. Worldwide 120–170 million persons are currently chronically Hepatitis C Virus (HCV) infected [2]. Due to repetitive and continuous inflammation, these patients are at increased risk of developing cirrhosis, subsequent liver decompensation and/or hepatocellular carcinoma. However, the current standard of care; pegylated interferon and rivabirin combination therapy is unsatisfied in the patients with high titre of HCVRNA and genotype 1b. Activated human liver stellate cells (HSC) with chronic viral infection, can play a pivotal role in the progression of liver fibrosis [3]. Activated HSC produce a number of profibrotic cytokines and growth factors that perpetuate the fibrotic process through paracrine and autocrine effects.

MicroRNAs (miRNAs) are endogenous small non-coding RNAs that control gene expression by degrading target mRNA or suppressing their translation [4]. There are currently 940 identifiable human miRNAs (The miRBase Sequence Database - Release ver. 15.0). miRNAs can recognize hundreds of target genes with incomplete complementary; over one third of human genes appear to be conserved miRNA targets [5] [6]. miRNA is associated several pathophysiologic events as well as fundamental cellular processes such as cell proliferation and differentiation. Aberrant expression of miRNA can be associated with the liver diseases [7] [8] [9] [10]. Recently reported miRNAs can regulate the activation of HSCs and thereby regulate liver fibrosis. miR-29b, a negative regulator for the type I collagen and SP1, is a key regulator of liver fibrosis [11]. miR-27a and 27b allowed culture-activated rat HSCs to switch to a more quiescent HSC phenotype, with restored cytoplasmic lipid droplets and decreased cell proliferation [12].

In this study, we aimed to reveal the association between miRNA expression patterns and the progression of liver fibrosis by using a chronic liver inflammation model in mouse. We also sought to identify the miRNA expression profile in chronic hepatitis (CH) C patients according to the degree of liver fibrosis, and to clarify how miRNAs contribute to the progression of liver fibrosis. We observed a characteristic miRNA expression profile common to both human liver biopsy specimens and mouse CCL_4_ specimens, comprising the key miRNAs which are associated with the liver fibrosis. This information is expected to uncover the mechanism of liver fibrosis and to provide a clearer biomarker for diagnosis of liver fibrosis as well as to aid in the development of more effective and safer therapeutic strategies for liver fibrosis.

## Results

### The expression level of several mouse miRNAs was increased by introducing mouse liver fibrosis

**Figure 1:**
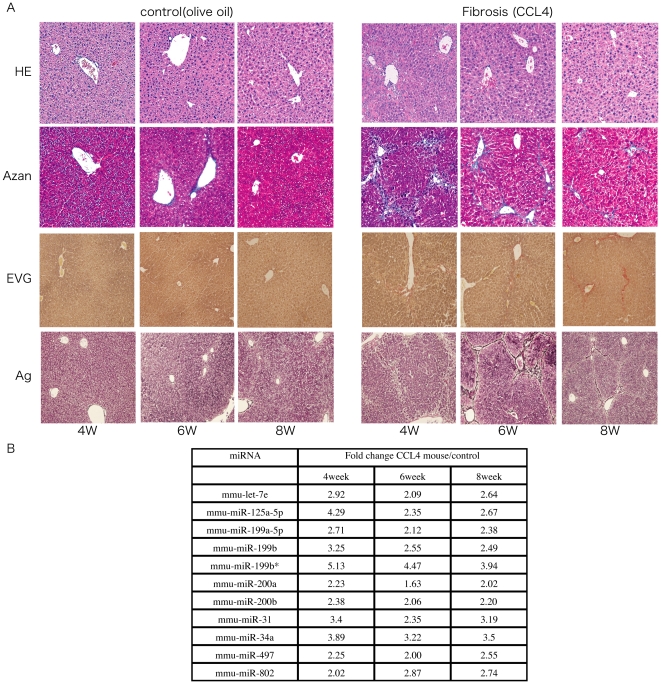
The change of liver fibrosis in mouse model. A. Representative H&E-stained, Azan-stained, Ag-stained, and EVG-stained histological sections of liver from mice receiving olive oil alone or CCL_4_ in olive oil. Magnification is ×10. B. The expression level of mmu-miRNA in mouse liver with olive oil or CCL_4_ at 4W, 6W, and 8W respectively, by microarray analysis.

In order to identify changes in the miRNA expression profile between advanced liver fibrosis and non-fibrotic liver, we intra-peritoneally administered CCL_4_ in olive oil or olive oil alone twice a week for 4 weeks and then once a week for the next 4 weeks. Mice were sacrificed at 4, 6, or 8 weeks and then the degree of mouse liver fibrosis was determined by microscopy (Figure S1). miRNA expression analysis was performed from the liver tissue collected at the same time. Histological examination revealed that the degree of liver fibrosis progressed in mice that received CCL_4_ relative to mice receiving olive oil alone (Figure 1A). Microarray analysis revealed that in CCL_4_ mice, the expression level of 11 miRNAs was consistently higher than that in control mice (Figure 1B).

### miRNA expression profile in each human liver fibrosis grade

**Figure 2:**
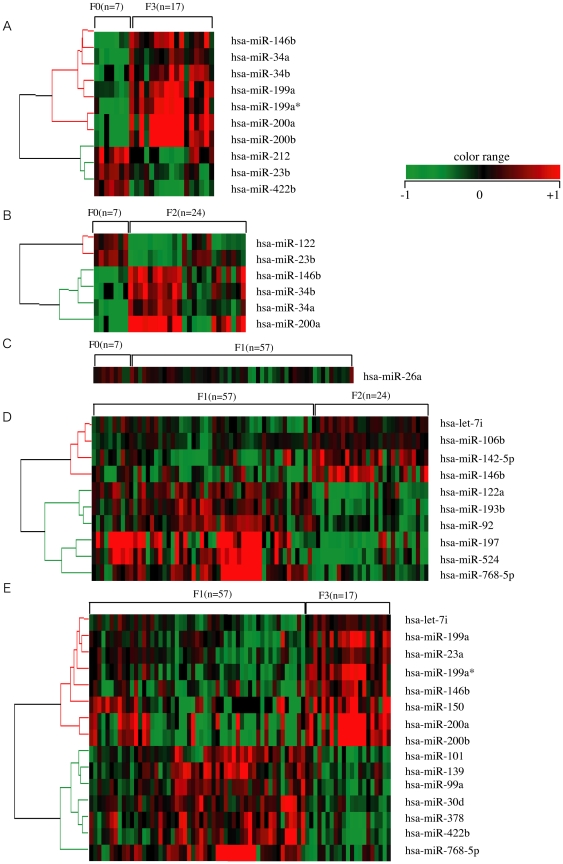
Liver fibrosis in human liver biopsy specimen. A. B. C. D. and E. miRNAs whose expression differs significantly between F0 and F3, F0 and F1, F0 and F2, F1 and F2, and F1 and F3, respectively. Relative expression level of each miRNA in human liver biopsy specimen by microarray. Data from microarray were also statistically analyzed using Welch's test and the Bonferroni correction for multiple hypotheses testing. Fold change, p-value are listed in Table S2.

We then established human miRNAs expression profile by using 105 fresh-frozen human chronic hepatitis (CH) C liver tissues without a history of anti-viral therapy, classified according to the grade of the liver fibrosis (F0, F1, F2, and F3 referred to METAVIR fibrosis stages)(Figure 2, Table S2). Fibrosis grade F0 was considered to be the negative control because these samples were derived from patients with no finding of liver fibrosis. In zebrafish, most highly tissue-specific miRNAs are expressed during embryonic development; approximately 30% of all miRNAs are expressed at a given time point in a given tissue [13]. In mammals, the 20–30% miRNA call rate has recently been validated [14]. Such analysis revealed that the diversity of miRNA expression level among specimens was small. Therefore, we focused on miRNAs with a fold change in mean expression level greater than 1.5 (p<0.05) in the two arbitrary groups of liver fibrosis.

Expression of several miRNAs was dramatically different among grades of fibrosis. In the mice study 11 miRNAs were related to the progression of liver fibrosis (mmu-let-7e, miR-125-5p, 199a-5p, 199b, 199b*, 200a, 200b, 31, 34a, 497, and 802). In the human study 10 miRNAs were extracted, and the change in their expression level varied significantly between F0 and F3 (F0<F3: hsa-miR-146b, 199a, 199a*, 200a, 200b, 34a, and 34b, F0>F3: hsa-miR-212, 23b, and 422b). The expression level of 6 miRNAs was significantly different between F0 and F2 (F0<F2: hsa-miR-146b, 200a, 34a, and 34b, F0>F2: hsa-miR-122 and 23b). 5 extracted miRNAs had an expression level that was significantly different between F1 and F2 (F1<F2: hsa-miR-146b, F1>F2: hsa-miR-122, 197, 574, and 768-5p). The expression level of 9 miRNAs changed significantly between F1 and F3 (F1<F3: hsa-miR-146b, 150, 199a, 199a*, 200a, and 200b, F1>F3: hsa-miR-378, 422b, and 768-5p). The miRNAs related to liver fibrosis were extracted using two criteria: similar expression pattern in both the human and the mice specimens and shared sequence between human and mouse. We compared the sequences of mouse miRNAs as described on the Agilent Mouse MiRNA array Version 1.0 (miRbase Version 10.1) and human miRNAs as described on the Agilent Human MiRNA array Version 1.5 (miRbase Version 9.1). The sequences of mmu-miR-199a-5p, mmu-miR-199b, mmu-miR-199b, mmu-miR-200a, and mmu-miR-200b in mouse miRNA corresponded to the sequences of hsa-miR-199a, hsa-miR-199a*, hsa-miR-199a, hsa-miR-200a, and hsa-miR-200b in human miRNA, respectively (Table S3).

### Validation of the microarray result by real-time qPCR

**Figure 3:**
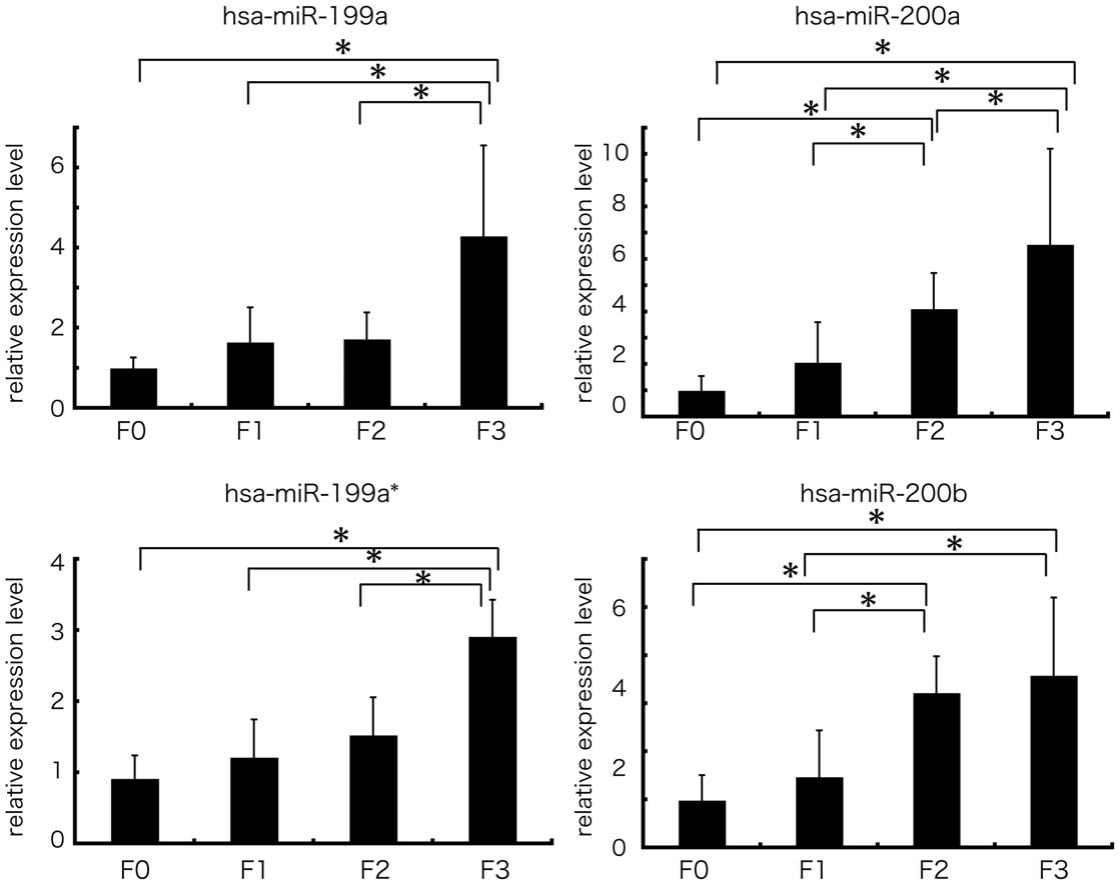
The expression level of miR-199 and 200 families in human liver biopsy specimen by real-time qPCR. Real-time qPCR validation of the 4 miRNAs (miR-199a, miR-199a*, miR-200a, and miR-200b). Each column represents the relative amount of miRNAs normalized to the expression level of U18. The data shown are the means+SD of three independent experiments. Asterisks indicates to a significant difference of p<0.05 (two-tailed Student-t test), respectively.

The 4 human miRNAs (miR-199a, miR-199a*, miR-200a, and miR-200b) with the largest difference in fold change between the F1 and F3 groups were chosen to validate the microarray results using stem-loop based real-time qPCR. The result of real-time qPCR supported the result of that microarray analysis. The expression level of these 4 miRNAs was significantly different between F0 and F3 and spearman correlation analysis also showed that the expressions of these miRNAs were strongly and positively correlated with fibrosis grade (n = 105, r = 0.498(miR-199a), 0.607(miR-199a*), 0.639(miR-200a), 0.618(miR-200b), p-values<0.0001) (Figure 3).

### Over expression of miR-199a, 199a*, 200a, and 200b was associated with the progression of liver fibrosis

**Figure 4:**
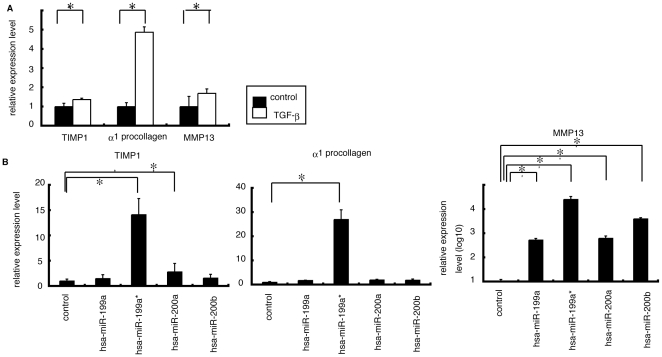
The relationship between expression level of miR-199 and 200 families and expression level of three fibrosis related genes. A. Administration of TGFβ in LX2 cells showed that the expression level of three fibrosis related genes were higher than that in non-treated cells. The data shown are the means+SD of three independent experiments. Asterisk was indicated to the significant difference of p<0.05 (two-tailed Student-t test). B. The expression levels of 3 fibrosis related genes in LX2 cells with overexpressing miR-199a, 199a*, 200a, or 200b, respectively were significantly higher than that in cells transfected with control miRNA (p<0.05; two-tailed Student t-test).

In order to reveal the function of miR-199a, miR-199a*, miR-200a, and miR-200b, we investigated the involvement of these miRNAs in the modulation of fibrosis-related gene in LX-2 cells. The endogenous expression level of these 4 miRNAs in LX2 and normal liver was low according to the microarray study (Figure S2). Transforming growth factor (TGF)β is one of the critical factors for the activation of HSC during chronic inflammation [15] and TGFβ strongly induced expression of three fibrosis-related genes include a matrix degrading complex comprised of α1 procollagen, matrix remodeling complex, comprised of metalloproteinases-13 (MMP-13), tissue inhibitors of metalloproteinases-1 (TIMP-1) in LX-2 cells (Figure 4A). Furthermore, overexpression of miR-199a, miR-199a*, miR-200a and miR-200b in LX-2 cells resulted significant induction of above fibrosis-related genes compared with control miRNA (Figure 4B). Finally we validated the involvement of TGFβ in the modulation of these miRNAs. In LX-2 cells treated with TGFβ, the expression levels of miR-199a and miR-199a* were significantly higher than in untreated cells; the expression levels of miR-200a and miR-200b were significantly lower than in untreated cells. Thus, our in vitro analysis suggested a possible involvement of miR-199a, 199a*, 200a, and 200b in the progression of liver fibrosis.

## Discussion

Our comprehensive analysis showed that the aberrant expression of miRNAs was associated with the progression of liver fibrosis. We identified that 4 highly expressed miRNAs (miR-199a, miR-199a*, miR-200a, and miR-200b) that were significantly associated with the progression of liver fibrosis both human and mouse. Coordination of aberrant expression of these miRNAs may contribute to the progression of liver fibrosis.

Prior studies have discussed the expression pattern of miRNA found in liver fibrosis samples between previous and present study. In this report and prior mouse studies and the expression pattern of 3 miRNAs (miR-199a-5p, 199b*, 125-5p) was found to be similar while the expression pattern of 11 miRNAs (miR-223, 221, 24, 877, 29b, 29a, 29c, 30c, 365, 148a, and 193) was partially consistent with fibrosis grade [16]. In low graded liver fibrosis, the low expression pattern of 3 miRNAs (miR-140, 27a, and 27b) and the high expression pattern of 6 miRNAs in rat miRNAs (miR-29c*, 143, 872, 193, 122, and 146) in rat miRNA was also similar to our mouse study (GEO Series accession number GSE19865) [11] [12] [17].

The results in this study and previously completed human studies reveal that the expression level of miR-195, 222, 200c, 21, and let-7d was higher in high graded fibrotic liver tissue than in low graded fibrotic liver tissue. Additionally, the expression level of miR-301, 194, and 122 was lower in the high graded fibrotic liver tissue than in low graded fibrotic liver tissue [18] [19] [20](GEO Series accession number GSE16922). This difference in miRNA expression pattern may be contributed to (1) the difference of microarray platform, (2) difference of analytic procedure, and (3) the difference of the species (rat, mouse, and human).

The miR-199 and miR-200 families have are circumstantially related to liver fibrosis. TGFβ-induced factor (TGIF) and SMAD specific E3 ubiquitin protein ligase 2 (SMURF2), both of which play roles in the TGFβ signaling pathway, are candidate targets of miR-199a* and miR-200b, respectively, as determined by the Targetscan algorithm. The expression of miR-199a* was silenced in several proliferating cell lines excluding fibroblasts [21]. Down regulation of miR-199a, miR-199a* and 200a in chronic liver injury tissue was associated with the hepatocarcinogenesis [9]. miR-199a* is also one of the negative regulators of the HCV replication [22]. According to three target search algorithms (Pictar, miRanda, and Targetscan), the miRNAs that may be associated with the liver fibrosis can regulate several fibrosis-related genes (Table S4). Aberrant expression of these miRNAs may be closely related to the progress of the chronic liver disease.

Epithelial-mesenchymal transition (EMT) describes a reversible series of events during which an epithelial cell loses cell-cell contacts and acquires mesenchymal characteristics [23]. Although EMT is not a common event in adults, this process has been implicated in such instances as wound healing and fibrosis. Recent reports showed that the miR-200 family regulated EMT by targeting EMT accelerator ZEB1 and SIP1 [24]. From our observations, overexpression of miR-200a and miR-200b can be connected to the progression of liver fibrosis.

The diagnosis and quantification of fibrosis have traditionally relied on liver biopsy, and this is still true at present. However, there are a number of drawbacks to biopsy, including the invasive nature of the procedure and inter-observer variability. A number of staging systems have been developed to reduce both the inter-observer variability and intra-observer variability, including the METAVIR, the Knodell fibrosis score, and the Scheuer score. However, the reproducibility of hepatic fibrosis and inflammatory activity is not as consistent [25]. In fact, in our study, the degree of fibrosis of the two arbitrary fibrosis groups was classified using the miRNA expression profile with 80% or greater accuracy (data not shown). Thus, miRNA expression can be used for diagnosis of liver fibrosis.

In this study we investigated whether common miRNAs in human and mouse could influence the progression of the liver fibrosis. The signature of miRNAs expression can also serves as a tool for understanding and investigating the mechanism of the onset and progression of liver fibrosis. The miRNA expression profile has the potential to be a novel biomarker of liver fibrosis. Moreover miRNA expression profiling has further applications in novel anti-fibrosis therapy in CH.

## Materials and Methods

### Sample preparation

105 liver tissues samples from chronic hepatitis C patients (genotype 1b) were obtained by fine needle biopsy (Table S1). METAVIR fibrosis stages were F0 in 7 patients, F1 in 57, F2 in 24 and F3 in 17. Patients with autoimmune hepatitis or alcoholic liver injury were excluded. None of the patients were positive for hepatitis B virus associated antigen/ antibody or anti human immunodeficiency virus antibody. No patient received interferon therapy or immunomodulatory therapy prior to the enrollment in this study. We also obtained normal liver tissue from the Liver Transplantation Unit of Kyoto University. All of the patients or their guardians provided written informed consent, and Kyoto University Graduate School and Faculty of Medicine's Ethics Committee approved all aspects of this study in accordance with the Helsinki Declaration.

### RNA preparation and miRNA microarray

Total RNA from cell lines or tissue samples was prepared using a *mir*Vana miRNA extraction Kit (Ambion, Austin, TX, USA) according to the manufacturer's instruction. miRNA microarrays were manufactured by Agilent Technologies (Santa Clara, CA, USA) and 100 ng of total RNA was labeled and hybridized using the Human microRNA Microarray Kit protocol for use with Agilent microRNA microarrays Version 1.5 and Mouse microRNA Microarray Kit protocol for use with Agilent microRNA microarrays Version 1.0. Hybridization signals were detected with a DNA microarray scanner G2505B (Agilent Technologies) and the scanned images were analyzed using Agilent feature extraction software (v9.5.3.1). Data were analyzed using GeneSpring GX 7.3.1 software (Agilent Technologies) and normalized as follows: (i) Values below 0.01 were set to 0.01. (ii) In order to compare between one-color expression profile, each measurement was divided by the 75th percentile of all measurements from the same species. The data presented in this manuscript have been deposited in NCBI's Gene Expression Omnibus and are accessible through GEO Series accession number GSE16922 (human) and accession number GSE19865 (mouse).

### Real-time qPCR for human miRNA

For detection of the miRNA level by real-time qPCR, TaqMan® microRNA assay (Applied Biosystems) was used to quantify the relative expression level of miR-199a (assay ID. 002304), miR-199a* (assay ID. 000499), miR-200a (assay ID. 000502), miR-200b (assay ID. 002251), and U18 (assay ID. 001204) was used as an internal control. cDNA was synthesized using the Taqman miRNA RT Kit (Applied Biosystems). Total RNA (10 ng/ml) in 5ml of nuclease free water was added to 3 ml of 5× RT primer, 10× 1.5µl of reverse transcriptase buffer, 0.15 µl of 100 mM dNTP, 0.19 µl of RNase inhibitor, 4.16 µl of nuclease free water, and 50U of reverse transcriptase in a total volume of 15 µl. The reaction was performed for 30 min at 16°C, 30 min at 42°C, and 5 min at 85°C. All reactions were run in triplicate. Chromo 4 detector (BIO-RAD) was used to detect miRNA expression.

### Animal and Chronic Mouse Liver Injury Model

Each 5 adult (8-week-old) male C57BL/6J mice were given a biweekly intra-peritoneal dose of a 10% solution of CCL_4_ in olive oil (0.02 ml/g/ mouse) for the first 4 weeks and then once a week for the next 4 weeks. At week 4, 6 or 8, the mice were sacrificed. Partial livers were fixed, embedded in paraffin, and processed for histology. Serial liver sections were stained with hematoxylin-eosin, Azan staining, Silver (Ag) staining, and Elastica van Gieson (EVG) staining, respectively. Total RNA from mice liver tissue was prepared as described previously. All animal procedures concerning the analysis of liver injury were performed in following the guidelines of the Kyoto University Animal Research Committee and were approved by the Ethical Committee of the Faculty of Medicine, Kyoto University.

### Cell lines and Cell preparation

The human stellate cell lines LX-2, was provided by Scott L. Friedman. LX-2 cells, which viable in serum free media and have high transfectability, were established from human HSC lines [26]. LX-2 cells were maintained in D-MEM (Invitrogen, Carlsbad, CA, USA) with 10% fetal bovine serum, plated in 60 mm diameter dishes and cultured to 70% confluence. Huh-7 and Hela cells were also maintained in D-MEM with 10% fetal bovine serum. HuS-E/2 immortalized hepatocytes were cultured as described previously [27]. LX-2 cells were then cultured in D-MEM without serum with 0.2% BSA for 48 hours prior to TGFβ1 (Sigma-Aldrich, Suffolk, UK) treatment (2.5 ng/ml for 20 hours). Control cells were cultured in D-MEM without fetal bovine serum.

### miRNA transfection

LX-2 cells were plated in 6-well plates the day before transfection and grown to 70% confluence. Cells were transfected with 50 pmol of Silencer® negative control siRNA (Ambion) or double-stranded mature miRNA (Hokkaido System Science, Sapporo, Japan) using lipofectamine RNAiMAX (Invitrogen). Cells were harvested 2 days after transfection.

### Real-time qPCR

cDNA was synthesized using the Transcriptor High Fiderity cDNA synthesis Kit (Roche, Basel, Switzerland). Total RNA (2 µg) in 10.4 µl of nuclease free water was added to 1 µl of 50mM random hexamer. The denaturing reaction was performed for 10min at 65°C. The denatured RNA mixture was added to 4 µl of 5× reverse transcriptase buffer, 2 µl of 10 mM dNTP, 0.5 µl of 40U/µl RNase inhibitor, and 1.1 µl of reverse transcriptase (FastStart Universal SYBR Green Master (Roche) in a total volume of 20 µl. The reaction ran for 30 min at 50°C (cDNA synthesis), and five min at 85°C (enzyme denaturation). All reactions were run in triplicate. Chromo 4 detector (BIO-RAD, Hercules, CA, USA) was used to detect mRNA expression. The primer sequences are follows; MMP13 s; 5′-gaggctccgagaaatgcagt-3′, as; 5′-atgccatcgtgaagtctggt-3′, TIMP1 s; 5′-cttggcttctgcactgatgg-3′, as; 5′-acgctggtataaggtggtct-3′, α1-procollagen s; 5′-aacatgaccaaaaaccaaaagtg-3′, as; 5′-cattgtttcctgtgtcttctgg-3′, and β-actin s; 5′-ccactggcatcgtgatggac-3′, as; 5′-tcattgccaatggtgatgacct-3′. Assays were performed in triplicate, and the expression levels of target genes were normalized to expression of the β-actin gene, as quantified using real-time qPCR as internal controls.

### Statistical analyses

Statistical analyses were performed using Student's *t*-test; *p* values less than 0.05 were considered statistically significant. Microarray data were also statistically analyzed using Welch's test and Bonferroni correction for multiple hypotheses testing.

## Supporting Information

Figure S1 Time line of the induction of chronic liver fibrosis. Upward arrow indicated administration of olive oil or CCL_4_. Downward arrow indicates when mice were sacrificed.
(TIF)

Figure S2 Comparison of the expression level of miR-199 and 200 familes in several cell lines and human liver tissue. Endogenous expression level of miR-199a, 199a*, 200a, and 200b in normal liver and LX2 cell as determined by microarray analysis (Agilent Technologies). Endogenous expression level of same miRNAs in Hela, Huh-7 and, immortalized hepatocyte: HuS-E/2 by previously analyzed data [9].
(TIF)

Table S1 Clinical characteristics of patients by the grade of fibrosis.
(DOCX)

Table S2 Extracted human miRNAs related to liver fibrosis.
(DOCX)

Table S3 Corresponding human and mouse miRNAs.
(DOCX)

Table S4 Hypothetical miRNA target genes according to in silico analysis.
(DOCX)
