# Supplementary figures and images for: The Progression of Liver Fibrosis Is Related with Overexpression of the miR-199 and 200 Families
Source: PLoS One. 2011 Jan 24;6(1):e16081. doi: 10.1371/journal.pone.0016081 (PMC3025920; doi:10.1371/journal.pone.0016081)

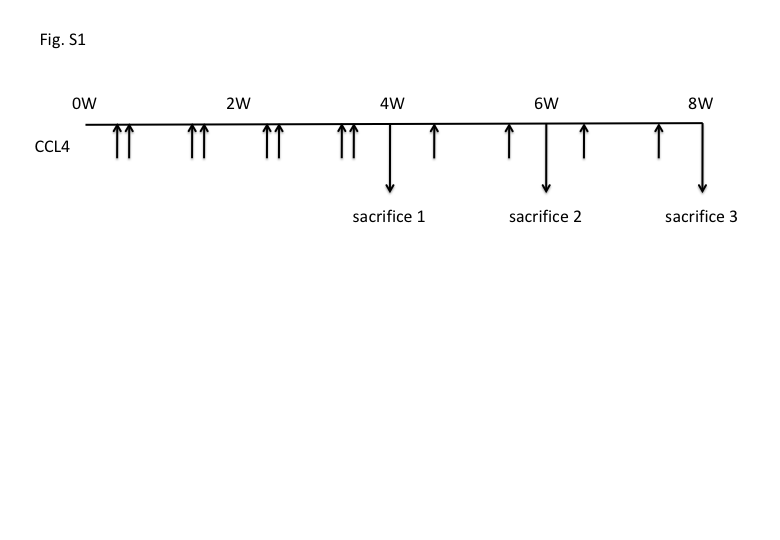

Supplement: Figure S1 — Time line of the induction of chronic liver fibrosis. Upward arrow indicated administration of olive oil or CCL4. Downward arrow indicates when mice were sacrificed. (TIF) [file pone.0016081.s001.tif]

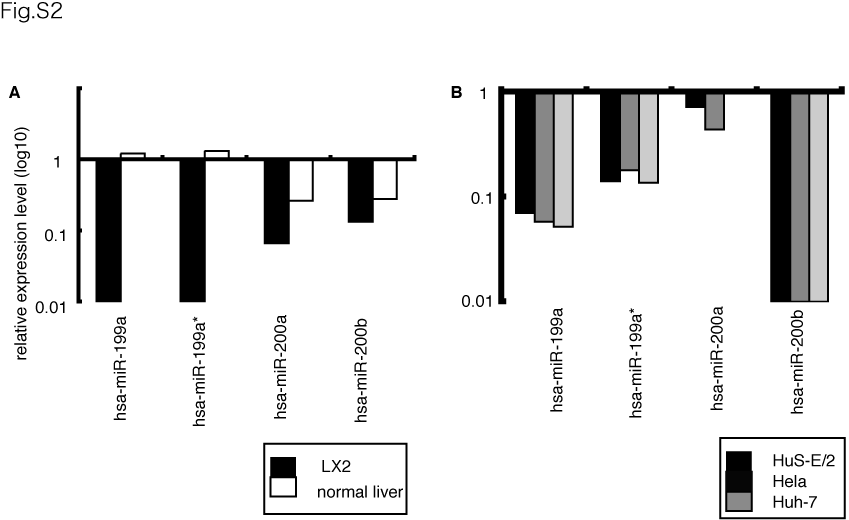

Supplement: Figure S2 — Comparison of the expression level of miR-199 and 200 familes in several cell lines and human liver tissue. Endogenous expression level of miR-199a, 199a*, 200a, and 200b in normal liver and LX2 cell as determined by microarray analysis (Agilent Technologies). Endogenous expression level of same miRNAs in Hela, Huh-7 and, immortalized hepatocyte: HuS-E/2 by previously analyzed data [9]. (TIF) [file pone.0016081.s002.tif]
