# Supplementary material for: The Progression of Liver Fibrosis Is Related with Overexpression of the miR-199 and 200 Families
Source: PLoS One. 2011 Jan 24;6(1):e16081. doi: 10.1371/journal.pone.0016081 (PMC3025920; doi:10.1371/journal.pone.0016081)
Supplement: Table S1 — Clinical characteristics of patients by the grade of fibrosis. (DOCX) [file pone.0016081.s003.docx]

Table S1. Clinical characteristics of patients by the grade of fibrosis

| Characteristics | F0 (*n*=7) | F1 (*n*=57) | F2 (*n*=24) | F3 (n=17) |
| --- | --- | --- | --- | --- |
| Age (years) | 43.3 ± 15.1 | 58.0 ± 9.3 | 60.0 ± 8.4 | 63.4 ± 5.1 |
| Male (%) | 4 (57%) | 28 (49%) | 14 (58%) | 7 (41%) |
| Weight (kg) | 60.6 ± 9.7 | 57.3 ± 8.6 | 59.1 ± 10.6 | 57.7 ± 7.4 |
| HCV RNA (x10^6^ copies/ml) | 1.12 ± 1.19 | 2.05 ± 1.60 | 2.06 ± 2.34 | 1.27 ± 0.76 |
| inflammatory grade |  |  |  |  |
| A0 | 2 | 0 | 0 | 0 |
| A1 | 5 | 59 | 3 | 0 |
| A2 | 0 | 7 | 21 | 6 |
| A3 | 0 | 0 | 0 | 11 |
| WBC(x10^3^/mm^3^) | 6.26 ± 1.31 | 5.16 ± 1.34 | 5.24 ± 1.41 | 4.97 ± 1.49 |
| Hemoglobin (g/dl) | 14.7 ± 2.06 | 14.0 ± 1.26 | 13.7 ± 1.37 | 13.9 ± 0.97 |
| Platelet (x10^4^/mm^3^) | 22.6 ± 4.5 | 17.3 ± 4.0 | 16.2 ± 5.5 | 12.1 ± 3.8 |
| AST (IU/L) | 57.0 ± 85.5 | 41.9 ± 0.42 | 70.5 ± 30.3 | 68.9 ± 27.6 |
| ALT (IU/L) | 63.1 ± 92.1 | 52.9 ± 73.5 | 86.6 ± 51.0 | 79.1 ± 47.5 |
| γGTP (IU/L) | 49.9 ± 55.9 | 46.1 ± 51.6 | 57.2 ± 33.7 | 77.3 ± 90.4 |
| ALP (IU/L) | 228 ± 54.7 | 239 ± 71.7 | 287 ± 89.0 | 338 ± 158.9 |
| Total bilirubin (mg/dl) | 0.67 ± 0.46 | 0.65 ± 0.24 | 0.70 ± 0.24 | 0.77 ± 30.3 |
| Albumin (g/dl) | 4.43 ± 0.35 | 4.24 ± 0.29 | 4.03 ± 0.36 | 3.97 ± 0.40 |

AST, aspartate aminotransferase; ALT, alanine aminotransferase; WBC, white blood cell; ALP, alkaline phosphatase; γGTP, gamma-glutamyl transpeptidase.
