# Supplementary material for: The Progression of Liver Fibrosis Is Related with Overexpression of the miR-199 and 200 Families
Source: PLoS One. 2011 Jan 24;6(1):e16081. doi: 10.1371/journal.pone.0016081 (PMC3025920; doi:10.1371/journal.pone.0016081)
Supplement: Table S2 — Extracted human miRNAs related to liver fibrosis. (DOCX) [file pone.0016081.s004.docx]

Table S2. Extracted human miRNAs related to liver fibrosis

| **F0 vs F3** | Gene Name | Fold Change (F0/F3) | p-value with FDR correction | p-value without any corrections |
| --- | --- | --- | --- | --- |
|  | **hsa-miR-199a** | **0.53** | **6.61E-04** | **1.94E-04** |
|  | **hsa-miR-422b** | **1.84** | **3.14E-03** | **1.17E-05** |
|  | **hsa-miR-146b** | **0.45** | **6.42E-03** | **4.98E-05** |
|  | **hsa-miR-212** | **2.26** | **8.23E-03** | **2.02E-03** |
|  | **hsa-miR-200a** | **0.14** | **9.61E-03** | **2.02E-04** |
|  | **hsa-miR-199a*** | **0.39** | **2.58E-02** | **4.29E-05** |
|  | **hsa-miR-34b** | **0.46** | **2.79E-02** | **2.68E-03** |
|  | **hsa-miR-200b** | **0.14** | **3.37E-02** | **3.61E-05** |
|  | **hsa-miR-34a** | **0.49** | **3.46E-02** | **2.03E-06** |
|  | **hsa-miR-23b** | **1.51** | **4.35E-02** | **2.60E-05** |
|  |  |  |  |  |
|  | hsa-miR-122a | 1.53 | 0.05< | 9.31E-04 |
|  |  |  |  |  |
| **F0 vs F1** | Gene Name | Fold Change (F0/F1) | p-value with FDR correction | p-value without any corrections |
|  | hsa-miR-26a | 1.21 | 3.54E-02 | 1.75E-03 |
|  |  |  |  |  |
|  | hsa-miR-122 | 1.08 | 0.05< | 4.55E-01 |
|  |  |  |  |  |
| **F0 vs F2** | Gene Name | Fold Change (F0/F2) | p-value with FDR correction | p-value without any corrections |
|  | **hsa-miR-146b** | **0.46** | **5.23E-03** | **2.66E-04** |
|  | **hsa-miR-122a** | **1.67** | **1.67E-02** | **2.46E-04** |
|  | **hsa-miR-23b** | **1.56** | **2.16E-02** | **5.00E-04** |
|  | **hsa-miR-200a** | **0.18** | **2.44E-02** | **3.11E-03** |
|  | **hsa-miR-34a** | **0.50** | **2.48E-02** | **1.58E-04** |
|  | **hsa-miR-34b** | **0.48** | **4.98E-02** | **4.09E-03** |
|  |  |  |  |  |
| **F1 vs F2** | Gene Name | Fold Change (F1/F2) | p-value with FDR correction | p-value without any corrections |
|  | **hsa-miR-197** | **2.06** | **1.74E-04** | **1.49E-04** |
|  | **hsa-miR-574** | **1.79** | **2.55E-04** | **4.18E-04** |
|  | **hsa-miR-768-5p** | **1.73** | **1.49E-03** | **3.97E-02** |
|  | **hsa-miR-122** | **1.51** | **2.30E-03** | **1.35E-06** |
|  | **hsa-miR-146b** | **0.64** | **1.28E-02** | **2.76E-06** |
|  | hsa-miR-193b | 1.46 | 1.19E-04 | 2.28E-07 |
|  | hsa-miR-92 | 1.35 | 1.26E-04 | 3.37E-06 |
|  | hsa-let-7i | 0.85 | 4.53E-02 | 9.80E-04 |
|  | hsa-miR-142-5p | 0.73 | 4.83E-02 | 6.69E-04 |
|  | hsa-miR-106b | 0.88 | 4.90E-02 | 8.88E-04 |
|  |  |  |  |  |
| **F1 vs F3** | Gene Name | Fold Change (F1/F3) | p-value with FDR correction | p-value without any corrections |
|  | **hsa-miR-200b** | **0.44** | **9.38E-05** | **3.55E-08** |
|  | **hsa-miR-200a** | **0.4** | **3.45E-04** | **6.03E-08** |
|  | **hsa-miR-199a*** | **0.57** | **4.16E-04** | **5.34E-10** |
|  | **hsa-miR-150** | **0.47** | **6.47E-04** | **3.68E-03** |
|  | **hsa-miR-199a** | **0.6** | **1.44E-03** | **2.99E-10** |
|  | **hsa-miR-146b** | **0.63** | **3.27E-03** | **1.30E-05** |
|  | **hsa-miR-422b** | **1.56** | **1.94E-02** | **1.46E-04** |
|  | **hsa-miR-768-5p** | **1.63** | **2.17E-02** | **9.50E-02** |
|  | **hsa-miR-378** | **1.57** | **4.87E-02** | **3.87E-05** |
|  | hsa-let-7i | 0.82 | 8.43E-04 | 2.69E-04 |
|  | hsa-miR-101 | 1.44 | 2.19E-03 | 1.05E-04 |
|  | hsa-miR-23a | 0.75 | 6.01E-03 | 1.51E-05 |
|  | hsa-miR-30d | 1.32 | 7.99E-03 | 1.96E-03 |
|  | hsa-miR-139 | 1.36 | 3.36E-02 | 8.52E-04 |
|  | hsa-miR-99a | 1.35 | 4.32E-02 | 2.10E-04 |
|  |  |  |  |  |
|  | hsa-miR-122 | 1.42 | 0.05< | 1.04E-04 |

The bold-face showed fold change <1.5 and p-value<0.05.
