# Supplementary material for: The Progression of Liver Fibrosis Is Related with Overexpression of the miR-199 and 200 Families
Source: PLoS One. 2011 Jan 24;6(1):e16081. doi: 10.1371/journal.pone.0016081 (PMC3025920; doi:10.1371/journal.pone.0016081)
Supplement: Table S3 — Corresponding human and mouse miRNAs. (DOCX) [file pone.0016081.s005.docx]

Table S3. Corresponding human and mouse miRNAs

| Agilent miRNA microarray | mouse v1.0 |  | human v1.5 |
| --- | --- | --- | --- |
| miRbase | mouse v10.1 | human v10.1 | Human v9.1 |
|  |  |  |  |
| miRNA  accession No. | >mmu-miR-199a-5p MIMAT0000229 | >hsa-miR-199a-5p MIMAT0000231 | >hsa-miR-199a MIMAT0000231 |
| sequence | CCCAGUGUUCAGACUACCUGUUC | CCCAGUGUUCAGACUACCUGUUC | CCCAGUGUUCAGACUACCUGUUC |
| miRNA  accession No. | >mmu-miR-199b MIMAT0004667 | >hsa-miR-199b-3p MIMAT0004563 | >hsa-miR-199a* MIMAT0000232 |
| sequence | ACAGUAGUCUGCACAUUGGUUA | ACAGUAGUCUGCACAUUGGUUA | ACAGUAGUCUGCACAUUGGUUA |
| miRNA  accession No. | >mmu-miR-199b* MIMAT0000672 | >hsa-miR-199b-5p MIMAT0000263 | >hsa-miR-199a MIMAT0000231 |
| sequence | CCCAGUGUUUAGACUACCUGUUC | CCCAGUGUUUAGACUAUCUGUUC | CCCAGUGUUCAGACUACCUGUUC |
| miRNA  accession No. | >mmu-miR-200a MIMAT0000519 | >hsa-miR-200a MIMAT0000682 | >hsa-miR-200a MIMAT0000682 |
| sequence | UAACACUGUCUGGUAACGAUGU | UAACACUGUCUGGUAACGAUGU | UAACACUGUCUGGUAACGAUGU |
| miRNA  accession No. | >mmu-miR-200b MIMAT0000233 | >hsa-miR-200b MIMAT0000318 | >hsa-miR-200b MIMAT0000318 |
| sequence | UAAUACUGCCUGGUAAUGAUGA | UAAUACUGCCUGGUAAUGAUGA | UAAUACUGCCUGGUAAUGAUGA |
