# Supplementary material for: The Progression of Liver Fibrosis Is Related with Overexpression of the miR-199 and 200 Families
Source: PLoS One. 2011 Jan 24;6(1):e16081. doi: 10.1371/journal.pone.0016081 (PMC3025920; doi:10.1371/journal.pone.0016081)
Supplement: Table S4 — Hypothetical miRNA target genes according to in silico analysis. (DOCX) [file pone.0016081.s006.docx]

Table S4. Hypothetical miRNA target genes according to in silico analysis

| **miRNA** | **Accession No.** | **Description** | **Gene symbol** |
| --- | --- | --- | --- |
| miR-200, miR-146 | NM_031370.2 | heterogeneous nuclear ribonucleoprotein D | AUF1 (*HNRPD*) |
| miR-146, miR-30 | NM_018014.3 | B-cell CLL/lymphoma 11A | BCL11A |
| miR-200b, miR-23, miR-30 | NM_022898.1 | B-cell CLL/lymphoma 11B | BCL11B |
| miR-30 | NM_000633.2 | B-cell CLL/lymphoma 2 | BCL2 |
| miR-101, **miR-30** | NM_138621.3 | BCL2-like 11 | BCL2L11 |
| miR-30 | NM_001706.3 | B-cell leukemia/lymphoma 6 | BCL6 |
| miR-139, miR-30 | NM_001123384.1 | BCL6 co-repressor | BCOR |
| miR-23 | NM_001167.2 | X-linked inhibitor of apoptosis | BIRC4 |
| miR-30 | NM_001127428.1 | caspase 3 | CASP3 |
| **miR-23** | NM_033340.2 | caspase 7 | CASP7 |
| miR-200b | NM_001025432.1 | CREB binding protein | CREBBP (*CBP*) |
| **miR-101,** miR-23 | NM_006565.2 | CCCTC-binding factor | CTCF |
| miR-23 | NM_001033883.1 | chemokine (C-X-C motif) ligand 12 | CXCL12 |
| **miR-199a*, miR-101,** miR-30 | NM_175629 | DNA (cytosine-5-)-methyltransferase 3 alpha | DNMT3A |
| miR-101 | NM_032482.2 | DOT1-like | DOT1L |
| miR-200b | NM_004417 | dual specificity phosphatase 1 | DUSP1 |
| miR-199a* | NM_004419 | dual specificity phosphatase 5 | DUSP5 |
| miR-199, miR-30 | NM_012199 | eukaryotic translation initiation factor 2C, 1 | EIF2C1 (AGO1) |
| miR-99 | NM_012154.2 | eukaryotic translation initiation factor 2C, 2 | EIF2C2 (AGO2) |
| miR-30 | NM_153402.1 | eukaryotic translation initiation factor 2C, 3 | EIF2C3 (AGO3) |
| miR-30 | NM_153177.2 | eukaryotic translation initiation factor 2C, 4 | EIF2C4 (AGO4) |
| miR-146 | NM_001419.2 | ELAV (embryonic lethal, abnormal vision, Drosophila)-like 1 | ELAVL1 (HUR) |
| miR-200a, miR-200b, miR-30 | NM_004432 | ELAV (embryonic lethal, abnormal vision, Drosophila)-like 2 | ELAVL2(Hel-N1) |
| miR-200b | NM_021952 | ELAV (embryonic lethal, abnormal vision, Drosophila)-like 4 | ELAVL4 |
| miR-200b | NM_001429 | E1A binding protein p300 | EP300(P300) |
| miR-101 | NM_152998.1 | enhancer of zeste homolog 2 | EZH2 |
| miR-23 | NM_174662.2 | Fas (TNF receptor superfamily, member 6) | FAS |
| miR-200, miR-139, miR-23 | NM_002024.4 | fragile X mental retardation 1 | FMRP(FMR1) |
| miR-199a* | NM_005252.3 | FBJ murine osteosarcoma viral oncogene homolog | FOS |
| miR-30 | NM_001009988.1 | forkhead box O3A | FOXO3A |
| miR-200a | NM_032682.4 | forkhead box P1 | FOXP1 |
| miR-199a* | NM_004958 | mechanistic target of rapamycin (serine/threonine kinase) | FRAP1 |
| miR-199 | NM_002093.2 | glycogen synthase kinase 3 beta | GSK3B |
| miR-200 | NM_006037.3 | histone deacetylase 4 | HDAC4 |
| miR-30 | NM_058176.2 | histone deacetylase 9 | HDAC9 |
| miR-199 | NM_001530.3 | hypoxia inducible factor 1, alpha subunit | HIF1A |
| miR-200, miR-146 | NM_031370.2 | heterogeneous nuclear ribonucleoprotein D | HNRPD |
| miR-23 | NM_000641.2 | interleukin 11 | IL11 |
| miR-30 | NM_000575.3 | interleukin 1, alpha | IL1A |
| miR-23 | NM_000565.2 | interleukin 6 receptor | IL6R |
| miR-146 | NM_001569.3 | interleukin-1 receptor-associated kinase 1 | IRAK1 |
| miR-23 | NM_002199.3 | interferon regulatory factor 2 | IRF2 |
| **miR-30** | NM_002460.2 | interferon regulatory factor 4 | IRF4 |
| miR-199a*, miR-30 | NM_018433.5 | lysine (K)-specific demethylase 3A | JMJD1A |
| miR-23 | NM_014663.2 | lysine (K)-specific demethylase 4A | JMJD2A |
| miR-200a | NM_002228 | jun oncogene | JUN/AP1 |
| miR-199a* | NM_002755 | mitogen-activated protein kinase kinase 1 | MAP2K1 |
| miR-199a* | NM_015093.2 | mitogen-activated protein kinase kinase kinase 7 interacting protein 2 (MAP3K7IP2) | MAP3K7IP2 |
| miR-30 | NM_004992.3 | methyl CpG binding protein 2 | MECP2 |
| miR-101, miR-30 | NM_005933.2 | myeloid/lymphoid or mixed-lineage leukemia | MLL |
| miR-23 | NM_003743.4 | nuclear receptor coactivator 1 | NCOA1 (*SRC1*) |
| miR-200b**, miR-199** | NM_006540 | nuclear receptor coactivator 2 | NCOA2(GRIP1) |
| miR-30 | NM_006312.3 | nuclear receptor co-repressor 2 | NCOR2 (*SMRT*) |
| miR-200b, | NM_002505.4 | nuclear transcription factor Y, alpha | NF-YA |
| miR-146, miR-139, miR-30 | NM_138714.2 | nuclear factor of activated T-cells | NFAT5 |
| miR-23 | NM_005417.3 | v-src sarcoma (Schmidt-Ruppin A-2) viral oncogene homolog | NOCA1(SRC1) |
| miR-30 | NM_002583.2 | PRKC, apoptosis, WT1, regulator | PAWR |
| miR-200b | NM_003884.4 | K(lysine) acetyltransferase 2B | PCAF |
| miR-101, mir-30 | NM_000945.3 | protein phosphatase 3 (formerly 2B), regulatory subunit B, alpha isoform | PPP3R1(Calcineurin B, type I) |
| miR-200b, miR23b, miR-30 | NM_001198.3 | PR domain-containing protein 1 beta | PRDM1 (*Blimp1*) |
| miR-200b, miR-150 | NM_002737 | protein kinase C, alpha | PRKCA |
| miR-200a, miR-101 | NM_005400 | protein kinase C, epsilon | PRKCE |
| miR-199a*, miR-200 | NM_006908.4 | ras-related C3 botulinum toxin substrate 1 | RAC1 |
| miR-101, miR-30, miR-200a, **miR-146** | NM_006908.4 | retinoic acid receptor, beta | RARB |
| miR-200b | NM_001664.2 | ras homolog gene family, member A | RHOA |
| **miR-30**, miR-200a, **miR-199** | NM_012238.4 | sirtuin (silent mating type information regulation 2 homolog) 1 | SIRT1 |
| miR-30 | NM_005900.2 | SMAD family member 1 | SMAD1 |
| miR-23 | NM_005902.3 | SMAD family member 3 | SMAD3 |
| miR-146 | NM_005359.5 | SMAD family member 4 | SMAD4 |
| miR-99 | NM_005904.2 | SMAD family member 7 | SMAD7 |
| miR-101 | NM_003069.3 | SWI/SNF related, matrix associated, actin dependent regulator of chromatin, subfamily a, member 1 | SMARCA1 |
| **miR-199a*** | NM_003070 | SWI/SNF related, matrix associated, actin dependent regulator of chromatin, subfamily a, member 2 | SMARCA2 (*BRM*) |
| miR-199a* miR-139 | NM_001128849 | SWI/SNF related, matrix associated, actin dependent regulator of chromatin, subfamily a, member 4 | SMARCA4 (*BRG1*) |
| **miR-99** | NM_003601.2 | SWI/SNF related, matrix associated, actin dependent regulator of chromatin, subfamily a, member 5 | SMARCA5 (ISWI) |
| miR-150 | NM_003075.3 | SWI/SNF related, matrix associated, actin dependent regulator of chromatin, subfamily c, member 2 | SMARCC2 |
| miR-150**, miR-199** | NM_003076.4 | SWI/SNF related, matrix associated, actin dependent regulator of chromatin, subfamily d, member 1 | SMARCD1 |
| **miR-30** | NM_001098426.1 | SWI/SNF related, matrix associated, actin dependent regulator of chromatin, subfamily d, member 2 | SMARCD2 |
| miR-200b | NM_022739.3 | SMAD specific E3 ubiquitin protein ligase 2 | SMURF2 |
| **miR-200a** | NM_003151.2 | signal transducer and activator of transcription 4 | STAT4 |
| miR-200a | NM_003152.2 | signal transducer and activator of transcription 5A | STAT5A |
| miR-23 | NM_012448.3 | signal transducer and activator of transcription 5B | STAT5B |
| **miR-199a*** | NM_003153.3 | signal transducer and activator of transcription 6, interleukin-4 induced (STAT6) | STAT6 |
| miR-199 | NM_004604.3 | syntaxin 4 | STX4 |
| **miR-101** | NM_004612.2 | transforming growth factor, beta receptor 1 | TGFBR1 |
| miR-23 | NM_003243.3 | transforming growth factor, beta receptor III | TGFBR3 |
| **miR-199a*, miR-23, miR-139** | NM_170695.2 | TGIF-interacting ubiquitin ligase 1 | TGIF |
| **miR-146** | NM_004620.2 | TNF receptor-associated factor 6 | TRAF6 |

Abbreviation: bold letter: common candidate from 3 algorithms (Pictar+ MiRanda+ Targetscan)
